# An app for mycetism in emergency care

**DOI:** 10.1515/almed-2020-0066

**Published:** 2020-08-08

**Authors:** Salvador Ventura Pedret, Esther Solé Llop, Josep M. Queraltó Compañó, Jaume-Miquel March-Amengual

**Affiliations:** Laboratori, Clínic Metropolitana Sud, Institut Català de la Salut, Comisión de Toxicología y Monitorización de Fármacos de la SEQC^ML^ , Barcelona, Spain; Hospital Clínico “Lozano Blesa” de Zaragoza, Salud Aragón, Comisión de Toxicología y Monitorización de Fármacos de la SEQC^ML^ , Zaragoza, Spain; Comisión de Toxicología y Monitorización de Fármacos de la SEQC^ML^ , Barcelona, Spain; Grupo de investigación en Metodología, Métodos, Modelos y Resultados de Salud y Ciencias, Sociales (M3O), Facultad de Ciencias de la Salud y Bienestar, Centrode Investigación en Salud y Asistencia Social (CESS), Universidad de Vic, Universidad Central de Cataluña (UVIC-UCC), Vic, Spain

**Keywords:** intoxications, mycetism, WhatsApp

To the Editor,

In the ’80s, a case was reported of intoxication for the ingestion of the fungus *Galerina sulciceps*, one of the most toxic species in the world. When ingested, the concentration of anatoxin causes a mortality of 75%, as compared to the estimated 30% of mortality associated with untreated intoxication with *Amanita phalloides*. Of note, *G. sulciceps* grows in Java and Ceylon. However, the case reported was not an isolated incident as two intoxications with *G. sulciceps* were documented in the city of Duyun, province of Guizhou, China, in November 2013 [1]. These reports suggest that this species is progressively spreading over the planet. *Clitocybe amoenolens* induces the restless legs syndrome caused by acromelic acid, which affects the metabolism of tryptophan in the myelinated fibers of the autonomic nervous system. Depending on the dose, it manifests in the form of edema, redness in fingers and toes, and very intense nocturnal paroxysmal pain. It is striking that this species, discovered in the Atlas Mountains of Morocco, has been found in Haute-Savoie, France [2], in Almarza de Cameros and Guadalajara, Spain [3], and in Italy [4]. The increase in the number of intoxications with *Podostroma cornu-damae* is also a matter of special concern. This species may be mistaken for young shoots of *Ganoderma lucidum*, which is found in the woods of the Iberian Peninsula and is known as *‘‘pipa’’* (pipe) for its shape. Although it is not edible, it is taken as an infusion in the Far East and has been known for its anti-cancer properties, although the scientific evidence available is scarce [5], [6]. *Podostroma* causes a characteristic severe intoxication induced by mycotoxins of the trichothecenes family: satratoxin, roridin, and verrucarin. These toxins inhibit protein synthesis in the ribosome by preventing the function of peptidyl transferase. Symptoms include skin shedding, fatigue, pancytopenia, and kidney failure. It is associated with a high mortality. In 2018, four cases were reported in Europe [7]. Other examples of invasive fungi include *Amanita smithiana* in the Balearic Islands, Spain, and *Macrolepiota venenata* (*Lepiota morganii*) imported in root balls of Nordic firs at the end of the 20th century [8]. Of note, there are no reports of the presence of *A. phalloides* in Argentina before 1949 [9].

These incidents illustrate the association between globalization and fungal intoxications. Movement of goods worldwide has caused these species to spread through exports of plants or wood to regions with a favorable climate, boosted by the lack of competitors and apparent similarity with edible native species. This phenomenon anticipates an increase in cases of mycetism in the future.

One of the characteristics of this type of poisoning is the late diagnosis, which added to the probable increase in imported syndromes, and the lack of knowledge of these fungi made it necessary to develop a tool for early diagnosis and optimization of therapies.

The resources provided by information and communication technologies in health care are especially useful in areas that require immediate action such as emergency care. According to a survey conducted by the *Interactive Advertising Bureau* on the use of social networks in the USA, up to 31% of the population used WhatsApp for professional purposes in 2016 [10]. Moreover, 84% of physicians had a smartphone, 38% had an iPad, and 59% used them in routine practice. Spain, with 30.5 million of WhatsApp users, ranks 9th in number of users, only below Germany and Italy, as reported in an eMarketer study in August 2019 (available at https://www.emarketer.com/newsroom/index.php/whatsapp-a-key-driver-of-mobile-messaging-growth/). As a result, the benefits of electronic mail have decreased in favor of mobile apps [11].

In this context, the SEQC^ML^ Work group on Toxicology and Drug Monitoring, in collaboration with the Department of Engineering of Universitat de Vic, has designed a mobile website, MicoApp, to help clinicians in early diagnosis of fungal intoxications [12]. It is a free-access, user-friendly app that can be downloaded at https://www.micoapp.uvic.cat and just requires the user to log in. The website informs visitors that the app is “intended for healthcare professionals” and ‘does not replace any diagnostic judgment issued by the treating physician. It is a diagnostic support tool.’ You can find the instructions for use and specifications of this app in the study by Ruiz et al [12].

Real-time diagnosis is crucial in case of intoxication. However, only 50% of patients go to ER when symptoms are alarming. Unfortunately, this translates into late diagnosis and poorer clinical outcomes. *MicoApp* is not only useful for consultation but also useful as an education tool as it can simulate numerous clinical settings, including mixed fungal intoxications. This way, in a real situation, this app enables accurate diagnosis in the shortest time possible. The use of simulators in medical training is gaining popularity. Future versions of this app may include AI elements such as neural networks, which make it especially useful for training and education. Future versions may also incorporate intoxications caused by abuse drugs, vegetal/animal substances, and medicines, among others.

Notably, MicoApp is not an alternative diagnostic procedure that replaces standard diagnostic procedures [13], [14].

## References

[1] Xiang H, Zhou Y, Zhou C, Lei S, Yu H, Wang Y (2018). Investigation and analysis of *Galerina sulciceps* poisoning in a canteen. Clin Toxicol.

[2] Moreau P-A, Courtecuisse R, Guez D, Garcin R, Neville P, Saviuc P (2001). Analyse taxinomique d’une espèce toxique: clitocybe amoenolens Malençon. Cryptog Mycolog.

[3] Martínez F, Martínez R, Meléndez A, Pérez Del Amo CM (2010). Clitocybe amoenolens. Primera cita para España. Bol Soc Micol Madr.

[4] Leonardi M, Crulli G, Pacione G, Recchia G (2002). Un’intossicazione collettiva da Clitocybe amoenolens riconducibile alla sindrome acromelalgica. Micol Veget Medit.

[5] Jin X, Ruiz Beguerie J, Sze DM, Chan GC (2016). *Ganoderma lucidum* (Reishi mushroom) for cancer treatment. Cochrane Database Syst Rev.

[6] Wu GS, Guo JJ, Bao JL, Li XW, Chen XP, Lu JJ (2013). Anti-cancer properties of triterpenoids isolated from *Ganoderma lucidum* - a review. Expet Opin Invest Drugs.

[7] Serés García L Revisión Podostroma -cornu-damae.

[8] Siquier JL, Salom JC, Constantino C (2012). Contribució al coneixement micològic de les Illes Balears (Espanya). Contribución al conocimiento micológico de las Islas Baleares (España). Rev Catalana Micol.

[9] Talamoni M, Cabrerizo S, Cari C, Diaz M, Ortiz de Rozas M, Sager I (2006). Intoxicación por Amanita phalloides, diagnóstico y tratamiento. Arch Argent Pediatr.

[10] Antevenio. (2016). Análisis del informe de 2016 del IAB sobre el uso de redes sociales.

[11] Centro de innovación BBVA Salud y redes sociales 26-7-2016.

[12] Ruiz C, March-Amengual JM, Reches G, Ventura S, Queraltó JM (2019). Una aplicación app para el diagnóstico de los micetismos. Rev Lab Clín.

[13] Carvalho LM, Carvalho F, de Lourdes Bastos M, Baptista P, Moreira N, Monforte AR (2014). Non-targeted and targeted analysis of wild toxic and edible mushrooms using gas chromatography-ion trap mass spectrometry. Talanta.

[14] Gicquel T, Lepage S, Fradin M, Tribut O, Duretz B, Morel I (2014). Amatoxins (α- and β-Amanitin) and phallotoxin (Phalloidin) analyses in urines using high-resolution accurate mass LC-MS technology. J Anal Toxicol.

